# The C-Terminal Region Mesd Peptide Mimics Full-Length Mesd and Acts as an Inhibitor of Wnt/β-Catenin Signaling in Cancer Cells

**DOI:** 10.1371/journal.pone.0058102

**Published:** 2013-02-28

**Authors:** Cuihong Lin, Wenyan Lu, Wei Zhang, Angelina I. Londoño-Joshi, Donald J. Buchsbaum, Guojun Bu, Yonghe Li

**Affiliations:** 1 Drug Discovery Division, Southern Research Institute, Birmingham, Alabama, United States of America; 2 Department of Pharmacy, The First Affiliated Hospital of Fujian Medical University, Fuzhou, People's Republic of China; 3 Department of Molecular and Cellular Pathology, The University of Alabama at Birmingham, Birmingham, Alabama, United States of America; 4 Department of Radiation Oncology, The University of Alabama at Birmingham, Birmingham, Alabama, United States of America; 5 Department of Neuroscience, Mayo Clinic, Jacksonville, Florida, United States of America; Vanderbilt University Medical Center, United States of America

## Abstract

While Mesd was discovered as a specialized molecular endoplasmic reticulum chaperone for the Wnt co-receptors LRP5 and LRP6, recombinant Mesd protein is able to bind to mature LRP5 and LRP6 on the cell surface and acts as a universal antagonist of LRP5/6 modulators. In our previous study, we found that the C-terminal region of Mesd, which is absent in sequences from invertebrates, is necessary and sufficient for binding to mature LRP6 on the cell surface. In the present studies, we further characterized the interaction between the C-terminal region Mesd peptide and LRP5/6. We found that Mesd C-terminal region-derived peptides block Mesd binding to LRP5 at the cell surface too. We also showed that there are two LRP5/6 binding sites within Mesd C-terminal region which contain several positively charged residues. Moreover, we demonstrated that the Mesd C-terminal region peptide, like the full-length Mesd protein, blocked Wnt 3A- and Rspodin1-induced Wnt/β-catenin signaling in LRP5- and LRP6- expressing cells, suppressed Wnt/β-catenin signaling in human breast HS578T cells and prostate cancer PC-3 cells, and inhibited cancer cell proliferation, although the full-length Mesd protein is more potent than its peptide. Finally, we found that treatment of the full-length Mesd protein and its C-terminal region peptide significantly increased chemotherapy agent Adriamycin-induced cytotoxicity in HS578T and PC-3 cells. Together, our results suggest that Mesd C-terminal region constitutes the major LRP5/6-binding domain, and that Mesd protein and its C-terminal region peptide have a potential therapeutic value in cancer.

## Introduction

The low density lipoprotein receptor-related protein-5 (LRP5) and LRP6 are two members of the expanding low density lipoprotein receptor (LDLR) family. The Frizzled (Fzd) receptors can respond to Wnt proteins only in the presence of the Wnt co-receptor LRP5 or LRP6 to activate the canonical β-catenin pathway. In the absence of Wnts, β-catenin is sequestered in a complex that consists of the adenomatous polyposis coli (APC) tumor suppressor, Axin, glycogen synthase kinase-3β (GSK3β), and casein kinase 1 (CK1). This complex formation induces the phosphorylation of β-catenin by CK1 and GSK3β, which results in the ubiquitination and subsequent degradation of β-catenin by the 26S proteasome. The action of this complex is inhibited upon binding of Wnt to its cell-surface receptors Fzd and LRP. The LRP-Wnt-Fzd binding results in stabilization of cytosolic β-catenin, which then enters the nucleus to form a complex with transcription factors of the T-cell factor/lymphoid enhancing factor (TCF/LEF) family to activate transcription of Wnt target genes that regulate cell cycle, growth, and progression [1]–[3].

LRP5 and LRP6 are subjected to modulation by several secreted proteins which bind to the extracellular β-propeller/EGF repeat modules of LRP5/6 [3]. Wnt and Rspondin (Rspo) proteins are two groups of Wnt/β-catenin signaling agonists. Similar to Wnt ligands, the action of Rspo proteins on Wnt/β-catenin signaling requires the Wnt receptors Fzd and LRP5/6, although the direct binding between Rspo and LRP5/6 is controversial [4]–[8]. Furthermore, Rspo proteins are able to synergize with Wnt ligands to activate Wnt/β-catenin signaling [5], [8], [9].

While Mesd was discovered as a specialized molecular endoplasmic reticulum (ER) chaperone for the Wnt co-receptors LRP5 and LRP6 [10], [11], recombinant Mesd protein is able to bind to mature LRP5 and LRP6 on the cell surface, acts as a universal inhibitor of different LRP5/6 modulators, and suppresses Wnt/β-catenin signaling in Wnt-dependent cancer cells [8], [12]–[14]. In our previous study, we found that the C-terminal region of Mesd, which is absent in sequences from invertebrates, is necessary and sufficient for binding to mature LRP6 on the cell surface [12]. In the present studies, we characterized the interaction between the C-terminal region Mesd peptide and LRP5/6. We also studied the role of the C-terminal region Mesd peptide in Wnt/β-catenin signaling in cancer cells.

## Materials and Methods

### Materials

Plasmid pcDNA3.1C-Myc-hLRP5 containing the full-length human LRP5 cDNA and plasmid pCS-Myc-hLRP6 containing the full-length human LRP6 cDNA were from Dr. Cindy Bartels (Case Western Reserve University, Cleveland) and Dr. Christof Niehrs (Deutsches Krebsforschungszentrum, Heidelberg, Germany), respectively. Plasmid pGST-E-cadherin was provided by Dr. Gail Johnson (University of Rochester, New YorK). Plasmid pUSEamp-Wnt1-HA containing the full-length mouse Wnt1 cDNA was purchased from Upstate. Plasmid BA-Wnt10b containing full-length mouse Wnt10b were from Addgene. The TOPFlash luciferase construct was from Upstate Biotechnology, and the Super8XTOPFlash luciferase construct was provided by Dr. Randall T. Moon (University of Washington, Seattle). A β-galactosidase-expressing vector was from Promega. Preparation of recombinant mouse Mesd protein and mouse Mesd C-terminal region peptide, mMesd (50–195), has been described before [12]. All other mouse Mesd peptides, human Mesd C-terminal region peptide hMesd (160–197) (KGGGSKEKNKTKQDKGKKKKEGDLKSRSSKEENRAGNK) and its control peptide (KEGDRKPRASKEGDRKPRASKEGDRKPRASKEGDRKPR) were manufactured by EZBiolab. Recombinant human Rspo1 protein was provided by Dr. Kyung-Ah Kim (Nuvelo Inc., California). Adriamycin was purchased from Sigma. Anti-osteoprotegerin (OPG) antibody was obtained from R&D Systems. Monoclonal anti-phosphorylated-LRP6 and anti-Axin2 were purchased from Cell Signaling Technology. Monoclonal anti-β-catenin was from BD Biosciences. Polyclonal rabbit anti-Cyclin D1 was from Chemicon International. Polyclonal anti-Wnt10b and monoclonal anti-actin was from Sigma. The monoclonal anti-HA antibody was generated as previously described [15]. Peroxidase labeled anti-mouse antibody and ECL system were purchased from Amersham Life Science. The luciferase and β-galactosidase assay systems were from Promega. Tissue culture media, fetal bovine serum (FBS), and plastic-ware were obtained from Life Technologies, Inc. Proteinase inhibitor cocktail Complete™ was obtained from Boehringer Mannheim. Proteins were iodinated by using the IODO-GEN method as described previously [8].

### Cell culture and conditioned media

Human Embryonic Kidney HEK 293 cells, human fibrosarcom cancer HT1080 cells, human basal-like breast cancer HS578T cells, human prostate cancer PC-3 cells, uncommitted mouse mesenchymal C2C12 cells, Wnt3A-secreting L cells, and control L cells were obtained from American Type Culture Collection. LDLR- deficient Chinese hamster ovary (CHO) cell line ldlA7 was kindly provided by Dr. Monty Krieger (Massachusetts Institute of Technology, Cambridge) [16]. LRP5-trasfected ldl-7 cells and the control cells have been described before [8], and were cultured in Ham's F-12 medium containing 10% of FBS and 350 µg/ml of G418. LRP6-transduced HT1080 cells and the control cells have been described before [17]. LRP6-transduced HT1080 and Wnt3A-secreting L cells were cultured in DMEM medium containing 10% of FBS and 350 µg/ml of G418. HEK293, HS578T and C2C12 cells were cultured in the same above medium without G418. PC-3 cells were cultured in RPMI-1640 medium containing 10% FBS. Wnt3A-conditioned medium (CM) and L cell control CM were prepared according to manufacturer's specifications.

### Ligand binding assay

Ligand binding assay was carried out exactly as previously described [8]. Cells (2×10^5^) were seeded into 12-well dishes 1 day prior to assay. Ligand-binding buffer (minimal Eagle's medium containing 0.6% BSA with a different concentration of radioligand, 0.6 ml/well) was added to cell monolayers, in the absence or the presence of unlabeled Mesd or its peptide, followed by incubation for 4 h at 4°C. Thereafter, overlying buffer containing unbound ligand was removed, and cell monolayers were washed and lysed in low-SDS lysis buffer (62.5 mM Tris-HCl pH 6.8, 0.2% SDS, 10% v/glycerol) and counted. The protein concentration of each cell lysate was measured in parallel dishes that did not contain the ligands.

### Ligand binding analysis

The AMBER9 simulation package [18] was used in both simulation and data processing. The peptides were represented using all-atom point-charge force field (AMBER ff03) [19]. A Generalized Born (GB) model [20] was used for all simulations with an effective 0.2 M salt concentration (modeled based on Debye-Hückel theory). In the GB method, the solvent is treated as a high-dielectric continuum interacting with charges that are embedded in solute molecules of lower dielectric environment. No surface area term was used in the GB model. The interior and solvent dielectric constants were set to 1.0 and 78.5, respectively. The temperature was controlled at 300 K using a weak-coupling scheme [21]. SHAKE [22] was applied to constrain all bonds connecting hydrogen atoms and a time step of 2.0 *fs* was used to numerically solve the Newtonian equations. The simulations of the two short peptides mMesd (160–169) and mMesd (183–191) were started from fully extended conformation after energy minimization and run for 100 *ns* each. The starting structure for peptide mMesd (155–191) was derived from the Mesd NMR structure (PDB ID: 2KGL) and the simulation was run for 200 *ns*. The simulated snapshots were clustered based on a hierarchical method [23] in which a snapshot was included into its closest cluster if the main-chain root-mean-square distance (RMSD) is smaller than 1.5 Å after sequential superimposition. The representative structures were compared, and the clusters whose representative structures were closer than 1.5 Å were merged together.

### Luciferase reporter assay

Cells were plated into 24-well plates. After overnight culture, the cells were transiently transfected with the TOPFlash or Super8XTOPFlash luciferase construct, β-galactosidase-expressing vector, and pcDNA3.1C-Myc-hLRP5, pCS-Myc-hLRP6 or control vector. After 24 h incubation, cells were treated with Mesd, Mesd peptide, Rspo1 and Wnt3A CM. Cells were then lysed 24 h later and both luciferase and β-galactosidase activities were determined. The luciferase activity was normalized to the β-galactosidase activity.

### Western blotting

Cells in 6-well plates were lysed in 0.5 ml of lysis buffer (phosphate-buffered saline containing 1% Triton X-100 and 1 mM PMSF) at 4°C for 10 min. Equal quantities of protein were subjected to SDS-PAGE under reducing conditions. Following transfer to immobilon-P transfer membrane, successive incubations with anti-HA, anti-Wnt10b, anti-β-catenin, anti-OPG, anti-phosphorylated-LRP6, anti-LRP6, anti-Axin2, anti-Cyclin D1 or anti-actin, and horseradish peroxidase-conjugated secondary antibody were carried out for 60–120 min at room temperature. The immunoreactive proteins were then detected using the ECL system. Films showing immunoreactive bands were scanned by Kodak Digital Science DC120 Zoom Digital Camera.

### Cytosolic free β-catenin analysis with GST-E-cadherin binding assay

The GST-E-cadherin binding assay was carried out exactly as previously described (*9*). Uncomplexed cytosolic free β-catenin present in 100 µg of total cell lysate was subjected to SDS-PAGE and detected using the monoclonal antibody to β-catenin.

### Cell proliferation assay

Cells were seeded into 6-well plates at a density of 10000 cells/well. RPMI-1640 or DMEM medium containing 2% FBS was used as assay media. After 16 h incubation, the cells were treated with Mesd or its peptide for 10 days. Media were changed every other day. At the end of the experiment, cells were harvested and counted using the trypan blue exclusion assay.

### Bromodeoxyuridine (BrdU) proliferation assay

BrdU incorporation into proliferating cells was analyzed by a cell proliferation ELISA BrdU kit (colorimetric, Roche) according to the manufacturer's instructions.

### Cell viability assay

Cell viability was measured in 96-well plates by the Cell Titer Glo Assay (Promega), which is a luminescent assay that is an indicator of live cells as a function of metabolic activity and ATP content.

### Statistics

Statistical analyses were performed using Student's unpaired t-test. Data were presented as mean ± SD.

## Results

### Mesd C-terminal region peptides block Mesd binding to LRP6 as well as LRP5 at the cell surface

**Figure 1:**
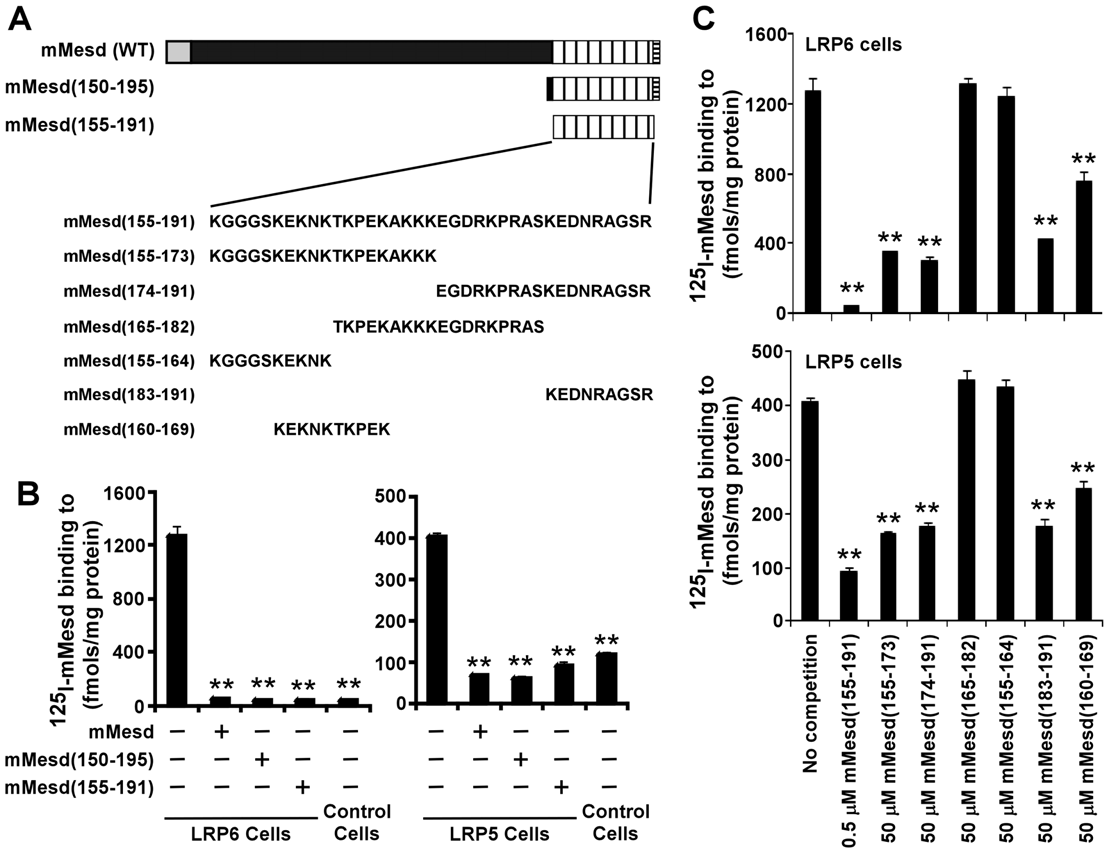
Mesd C-terminal region peptides block Mesd binding to LRP6 and LRP5 at the cell surface. (A) Schematic representation of mouse Mesd and its C-terminal region peptides and amino acid sequences of mouse Mesd C-terminal region peptides. (B) ^125^I-Mesd (5 nM) binding to LRP6-transduced HT1080 cells and the corresponding control cells was carried out for 4 h at 4°C in the absence or presence of 500 nM mouse Mesd or 500 nM mouse Mesd C-terminal region peptide. Values are the average of triple determinations with the s.d. indicated by error bars. (C) ^125^I-Mesd (5 nM) binding to LRP6-transduced HT1080 cells or LRP5-trasfected ldl-7 cells was carried out for 4 h at 4°C in the absence or presence of various mouse Mesd C-terminal region peptides at the indicated concentrations. Values are the average of triple determinations with the s.d. indicated by error bars. **P<0.01 compared to the LRP6 or LRP5 cells in the absence of Mesd and its peptides.

In our previous study, we have shown that the Mesd C-terminal region peptide mMesd (150–195) blocks the full-length Mesd protein binding to mature LRP6 at the cell surface [12]. However, whether the Mesd C-terminal region peptide is able to block Mesd protein binding to mature LRP5 at cell surface is unclear. The most part of the C-terminal region of Mesd is absent in sequences from invertebrates. As the first 5 amino acid residues at the N-terminus and the last 4 amino acid residues at the C-terminus of mMesd (150–195) are conserved across different species, we prepared a shorter mouse Mesd C-terminal region peptide, mMesd (155–191), which lacks these 9 amino acid residues (Figure 1A). We then performed ^125^I-Mesd binding assay with LRP6- and LRP5-expressing cells. We found that mMesd (155–191), like mMesd (150–195), blocked Mesd protein binding to LRP6 as well as LRP5 at the cell surface (Figure 1B), indicating that the Mesd C-terminal region is also important for Mesd binding to LRP5.

### There are two LRP5/6 binding sites within Mesd C-terminal region

To further characterize Mesd binding to LRP5/6, we prepared 6 Mesd peptides based on the mouse Mesd C-terminal region sequence (Figure 1A), and performed ^125^I-Mesd binding assay with LRP6- and LRP5-expressing cells in the presence or absence of these peptides. While mMesd (155–164) and mMesd (165–182) were unable to block Mesd protein binding to LRP5 or LRP6 at the cell surface, mMesd (160–169) showed a similar level of inhibition as mMesd (155–173) (Figure 1C). In addition, mMesd (183–191) also displayed a similar level of inhibition as mMesd (174–191) and mMesd (160–169) (Figure 1C). Together, these results indicate that there are two LRP5/6 binding sites within Mesd C-terminal region. Moreover, it is interestingly to note that mMesd (160–169) and mMesd (183–191) displayed their inhibitory ability at the concentration of 50 µM, which is 100 times of those required for mMesd (155–191), suggesting that the two LRP5/6 binding sites within Mesd C-terminal region cooperate with each other to create a high affinity binding domain to LRP5/6.

### Isolated C-terminal peptides maintain structural characteristics of the full-length Mesd

**Figure 2:**
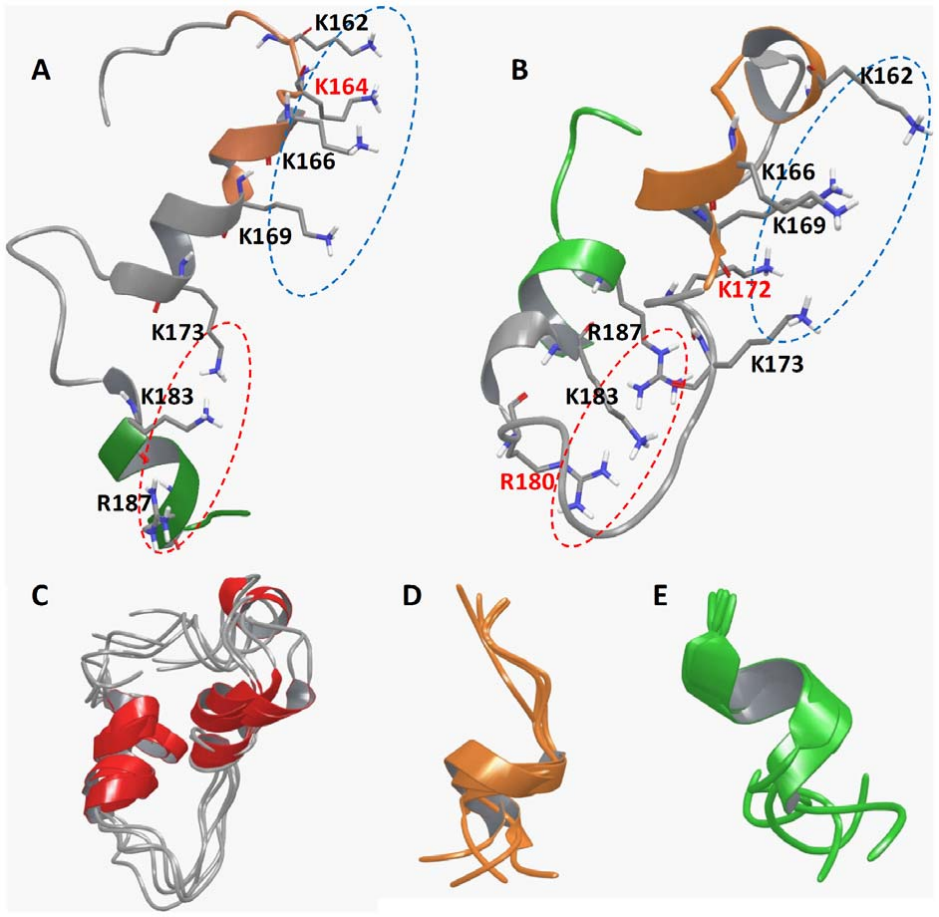
Structural representation of C-terminal peptides in cartons. (A) The NMR structure of mouse Mesd C-terminal domain (155–191) based on the study of Chen et al. (24). (B) The most representative snapshot of the peptide mMesd (155–191). The segments of 160–169 and 183–191 were highlighted in orange and green colors. Residues that form the positive surface were shown in solid sticks. The two surface regions that consist of residues from 160–169 segment or from 183–191segment were marked with blue and red dashed circles. Residues that are unique in the positive surface of A) or B) were labeled with red-color. (C–E) The most populated conformations based on clustering analysis of the simulation snapshots of three C-terminal peptides mMesd (155–191) (C), mMesd (160–169) (D) and mMesd (183–191) (E).

Our results of binding assays are consistent with recent NMR study on the full-length Mesd [24], which shows that the C-terminal flexible helical domain (156–195) is structurally distinct from the core domain (1–155), and is responsible for the escort function of Mesd by binding to LRP5/6. Nevertheless, peptides can have significant structural changes when isolated from proteins. To further explore the structural insights, we performed molecular dynamics simulations on all three peptides [mMesd (155–191), mMesd (160–169) and mMesd (183–191)] and compared them with the NMR structure of the full-length Mesd protein. Clustering analysis of the simulation results indicated that all three peptides adopted relative stable conformation. The most populated conformations of the three peptides were illustrated in Figure 2C–2E. Instead of the extended loop-like shape of C-terminal domain in the full-length Mesd (Figure 2A), mMesd (155–191) folds into a more compact structure with both its C- and N-termini bending towards the center (Figure 2B). Despite the apparent structural differences, both structures maintained a positive surface consisted of positively charged residues with their sidechains locate at the same side and fully exposed to solvent (Figure 2A and 2B). Such positive surface is crucial for Mesd's bind capacity to mature LRP5/6. Interestingly, this positive surface can be spatially divided into two regions that mainly consist of the positively charged residues from mMesd (160–169) and mMesd (183–190), respectively (Figure 2A and 2B). Simulation results show that ∼45% and ∼58% of all simulation snapshots of mMesd (160–169) and Mesd (182–190), respectively, adopted similar conformations as their corresponding ones in the full-length Mesd structure (Figure 2D and 2E). The similar structural characteristics and positive surface explain that the two shorter peptides display a similar function on binding to LRP5/6.

### The Mesd C-terminal region peptide blocks Wnt/β-catenin signaling induced by Wnt3A and Rspo1 in LRP5- or LRP6-expressing HEK293 cells

**Figure 3:**
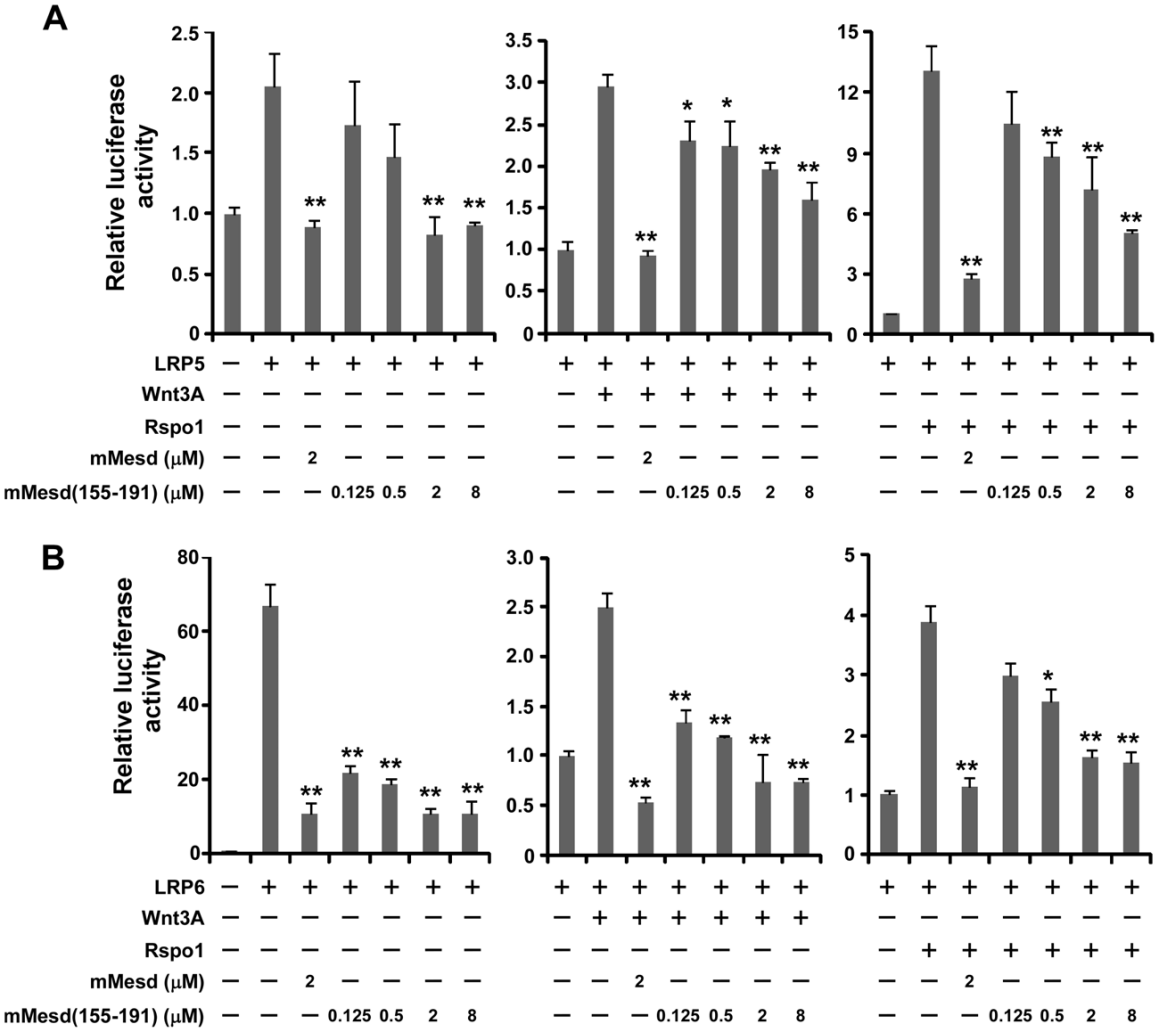
Mesd C-terminal region peptide blocks Wnt/β-catenin signaling induced by LRP5, LRP6, Wnt3A and Rspo1 in HEK293 cells. HEK293 cells in 24-well plates were transiently transfected with the LRP5 plasmid (A), the LRP6 plasmid (B) or the corresponding control vector, along with the TOPFlash luciferase construct and the β-galactosidase-expressing vector in each well. After 24 h incubation, cells were treated with Wnt3A CM (5%), Rspo1 (40 ng/ml), mouse Mesd (mMesd) (2 µM), or mouse Mesd C-terminal region peptide mMesd (155–191) at the indicated concentrations. The luciferase activity was then measured 24 h later with normalization to the activity of the β-galactosidase. Values are the average of triple determinations with the s.d. indicated by error bars. *P<0.05, **P<0.01 compared to the control cells without Mesd and its peptide treatment.

Wnt3A is one of the 19 vertebrate members of the Wnt family. There are four members in the Rspo family. Both Wnt3A and Rspo1 are able to activate Wnt/β-catenin signaling through LRP5 or LRP6 [5], [8], [9]. Therefore, we performed Wnt/β-catenin signaling reporter assay to test whether the Mesd C-terminal region peptide mimics Mesd protein to act as an inhibitor of Wnt/β-catenin signaling induced by Wnt3A and Rspo1 in LRP5- or LRP6-expressing cells. HEK293 cells were transiently transfected with LRP5 or LRP6 along with Wnt/β-catenin signaling reporter TOPFLash, and treated with Wnt3A CM or Rspo1 protein in the presence or absence of mouse Mesd protein or its C-terminal region peptide mMesd (155–191). As expected, TOPFlash activity was greatly increased after HEK293 cells were transiently transfected with LRP5 or LRP6 and treated with Wnt3A or Rspo1 (Figure 3). Importantly, the increased TOPFlash activity induced by LRP5, LRP6, LRP5 plus Wnt3A, LRP6 plus Wnt3A, LRP5 plus Rspo1, or LRP6 plus Rspo1 was blocked not only by Mesd protein but also by its peptide mMesd (155–191) in a dose dependent manner (Figure 3).

### The Mesd C-terminal region peptide blocks LRP6 phosphorylation and OPG expressioninduced by Wnt3A and Rspo1 in C2C12 cells

**Figure 4:**
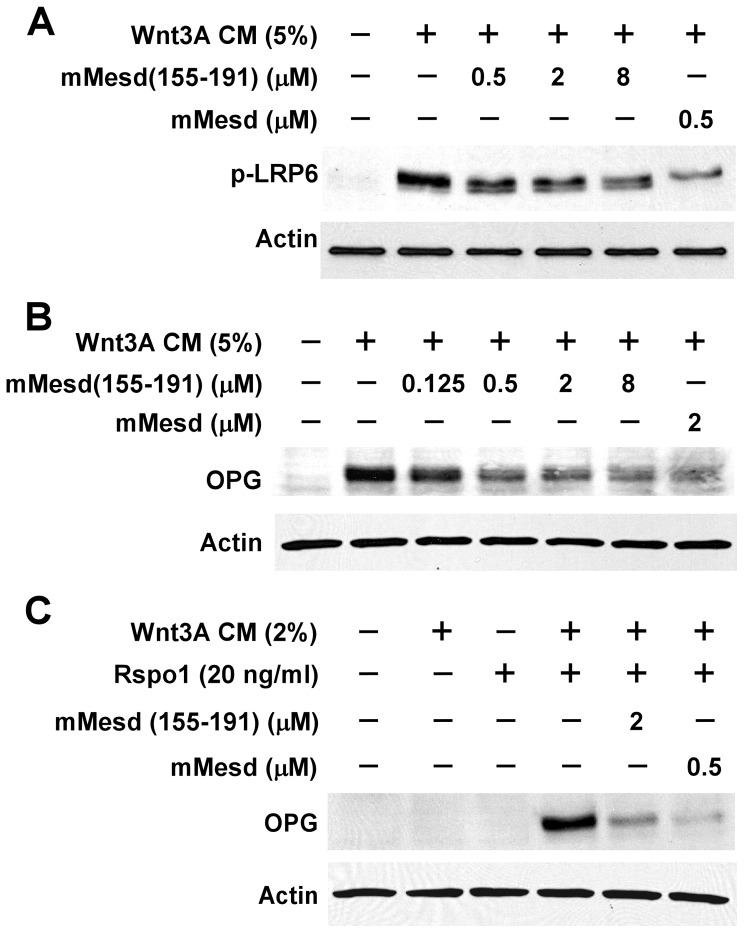
Mesd C-terminal region peptide blocks Wnt/β-catenin signaling induced by Wnt3A and Rspo1 in C2C12 cells. (A) C2C12 cells in 6-well plates were treated with Wnt3A CM (5%) in the absence or presence of mouse Mesd (mMesd) (0.5 µM) or mouse Mesd C-terminal region peptide mMesd (155–191) at the indicated concentrations for 6 h. The level of phosphorylated LRP6 was analyzed. (B) C2C12 cells in 6-well plates were incubated with Wnt3A CM (5%) in the presence or absence of mMesd (0.5 µM) or mMesd (155–191) at the indicated concentrations for 48 h. The levels of total cellular OPG were analyzed by Western blotting. (C) C2C12 cells in 6-well plates were incubated with Rspo1 (20 ng/ml) and/or Wnt3A CM (2%) in the absence or presence of mMesd (0.5 µM) or mMesd (155–191) (2 µM) for 48 h. The levels of total cellular OPG were analyzed by Western blotting. All the samples were also probed with the anti-actin antibody to verify equal loading.

C2C12 cells are uncommitted mouse mesenchymal progenitor cells that can be differentiated into osteoblasts upon the activation of Wnt/β-catenin signaling [9], [25], [26]. We employed C2C12 cells to further examine the effect of Mesd peptide on Wnt3A-induced Wnt/β-catenin signaling. LRP6 phosphorylation is critical for Wnt/β-catenin signaling induced by Wnt proteins [3], and OPG is a direct target gene of Wnt/β-catenin signaling in osteoblasts [27], [28]. We found that mouse Mesd C-terminal region peptide mMesd (155–191), like Mesd protein, was able to suppress Wnt3A-induced endogenous LRP6 phosphorylation and OPG expression in C2C12 cells (Figure 4A and 4B).

Rspo1 is able to synergizes with Wnt3A in inducing OPG expression in C2C12 cells, although Rspo1 itself has minor effects [9]. We found that mouse Mesd C-terminal region peptide mMesd (155–191), similar to Mesd protein, repressed OPG expression induced by Rspo1 plus Wnt3A in C2C12 cells (Figure 4C).

### The Mesd C-terminal region peptide attenuates Wnt/β-catenin signaling in prostate cancer PC-3 cells and breast cancer HS578T cells

**Figure 5:**
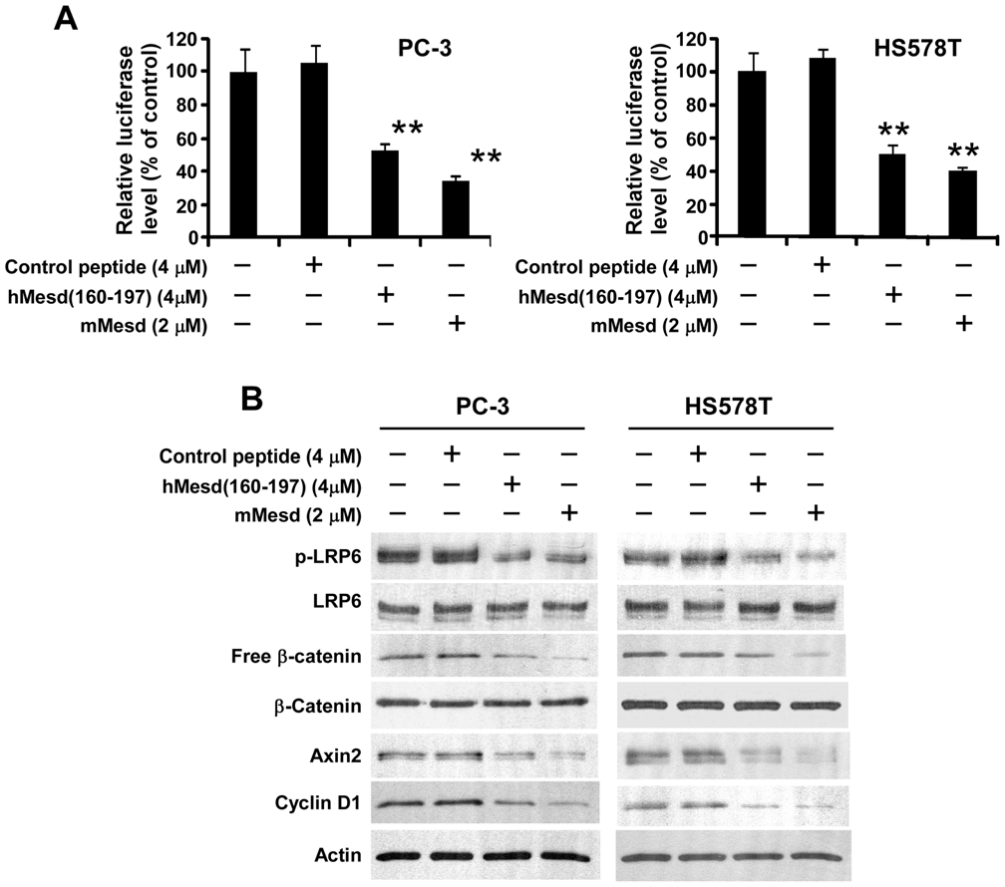
Mesd C-terminal region peptide blocks Wnt/β-catenin signaling in prostate cancer PC-3 and breast cancer HS578T cells. (A) PC-3 and HT578T cells in 24-well plates were transiently transfected with the Super8XTOPFlash luciferase construct and β-galactosidase-expressing vector in each well. After 24 h incubation, cells were treated with mouse Mesd (mMesd), human Mesd peptide hMesd (160–197) or control peptide at the indicated concentrations. The luciferase activity was then measured 24 h later with normalization to the activity of the β-galactosidase. Values are the average of triple determinations with the s.d. indicated by error bars. **P<0.01 compared to the control cells without Mesd or Mesd peptide treatment. (B) PC-3 and HS578T cells in 6-well plates were treated with mMesd, hMesd (160–197) or control peptide at the indicated concentrations for 24 h. The levels of cytosolic free β-catenin, and total cellular β-catenin, LRP6, Axin2, cyclin D1 and phosphorylated LRP6 were then analyzed by Western blotting. Samples were also probed with the anti-actin antibody to verify equal loading.

Accumulating evidence indicates that Wnt co-receptor LRP6 play a critical role in the Wnt/β-catenin signaling activation in human prostate and breast cancer cells and is important in the development and progression of these two types of cancer [8], [13], [14], [29]–[33]. To test whether the Mesd C-terminal region peptide is able to suppress Wnt/β-catenin signaling in human prostate and breast cancer cells, we prepared a human Mesd C-terminal region peptide hMesd (160–197) which is corresponding to mouse Mesd C-terminal region peptide mMesd (155–191). We also prepared a negative control peptide based on the sequence of mMesd (155–164). Similar to mMesd (155–191), hMesd (160–197) displayed inhibitory effects on Wnt/β-catenin signaling induced by LRP6, Wnt3A, Rspo1, Wnt1 and Wnt10b (Figure S1 and S2). We found that hMesd (160–197), but not the control peptide, mimicked Mesd protein and significantly repressed the Wnt reporter luciferase activity in prostate cancer PC3 cells and breast cancer HS578T cells (Figure 5A). Accordingly, hMesd (160–197) greatly decreased the levels of cytosolic free β-catenin, LRP6 phosphorylation and Wnt/β-catenin signaling targets Axin2 and cyclin D1 expression in PC-3 and HS578T cells (Figure 5B).

**Figure 6:**
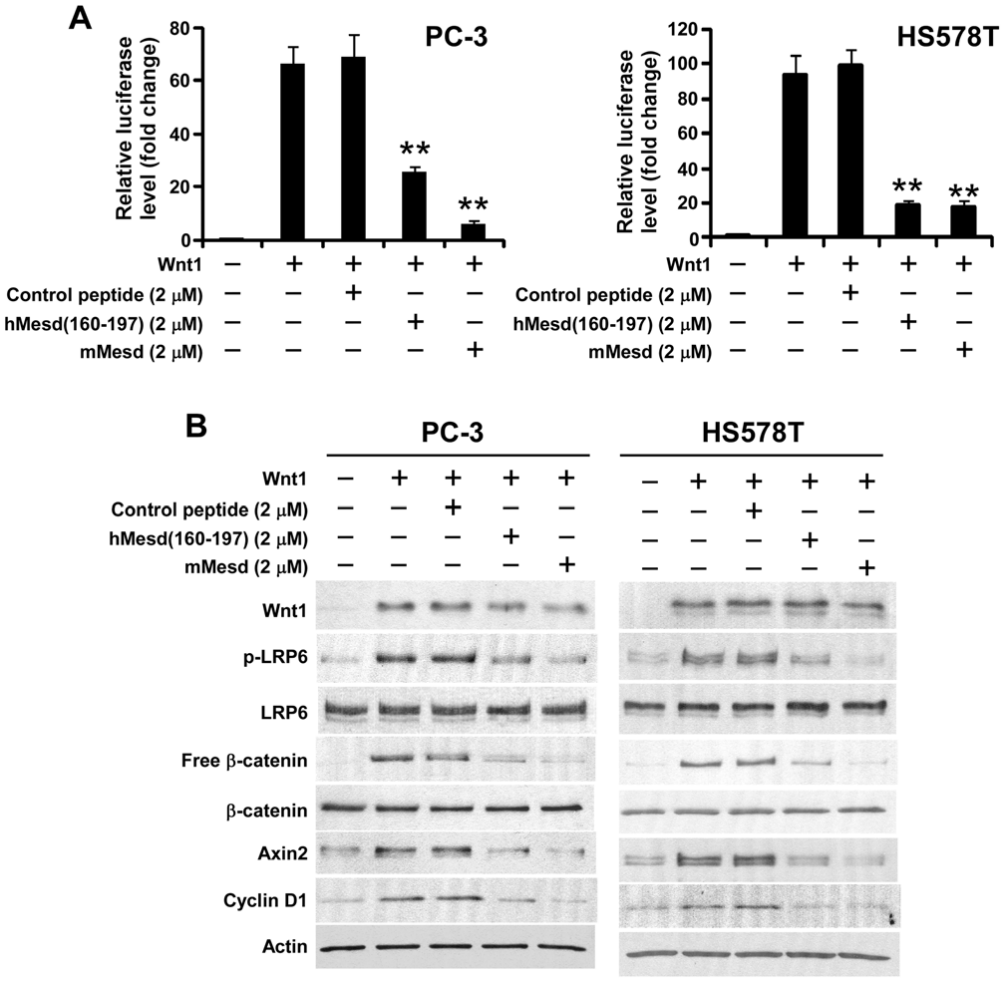
Mesd C-terminal region peptide blocks Wnt1-induced Wnt/β-catenin signaling in prostate cancer PC-3 and breast cancer HS578T cells. (A) PC-3 and HT578T cells in 24-well plates were transiently transfected with the Wnt1 plasmid along with the Super8XTOPFlash luciferase construct and β-galactosidase-expressing vector in each well. After 24 h incubation, cells were treated with mouse Mesd (mMesd), human Mesd peptide hMesd (160–197) or control peptide at the indicated concentrations. The luciferase activity was then measured 24 h later with normalization to the activity of the β-galactosidase. Values are the average of triple determinations with the s.d. indicated by error bars. **P<0.01 compared to the control cells without Mesd or Mesd peptide treatment. (B) PC-3 and HS578T cells in 6-well plates were transiently transfected with Wnt1 plasmid or the corresponding control vector. After being incubated for 24 h, cells were treated with mMesd, hMesd (160–197) or control peptide at the indicated concentrations for 24 h. The levels of cytosolic free β-catenin, and total cellular Wnt1, β-catenin, LRP6, Axin2, cyclin D1 and phosphorylated LRP6 were then analyzed by Western blotting. Samples were also probed with the anti-actin antibody to verify equal loading.

Wnt1 regulates cellular differentiation and proliferation of the mammary epithelium, and Wnt1 up-regulation in the mammary gland has been shown to induce mammary hyperplasia and adenocarcinoma [34], [35]. Overexpression of Wnt1 was found in the majority of prostate carcinoma specimens [36]. To further characterize the inhibitory effect of the Mesd C-terminal region peptide in prostate and breast cancer cells, we transiently transfected PC-3 and HS578T cells with Wnt1 and Wnt reporter plasmids and treated with Mesd protein or human Mesd peptide hMesd (160–197). We found again that hMesd (160–197), but not the control peptide, mimicked Mesd protein to significantly repress Wnt reporter luciferase activity induced by Wnt1 in PC3 and HS578T cells (Figure 6A). Moreover, hMesd (160–197) greatly decreased the levels of cytosolic free β-catenin, LRP6 phosphorylation and Wnt/β-catenin signaling targets Axin2 and cyclin D1 in Wnt1-expressing PC-3 and HS578T cells (Figure 6B).

### The Mesd C-terminal region peptide inhibits prostate cancer PC-3 and breast cancer HS578T cell proliferation

**Figure 7:**
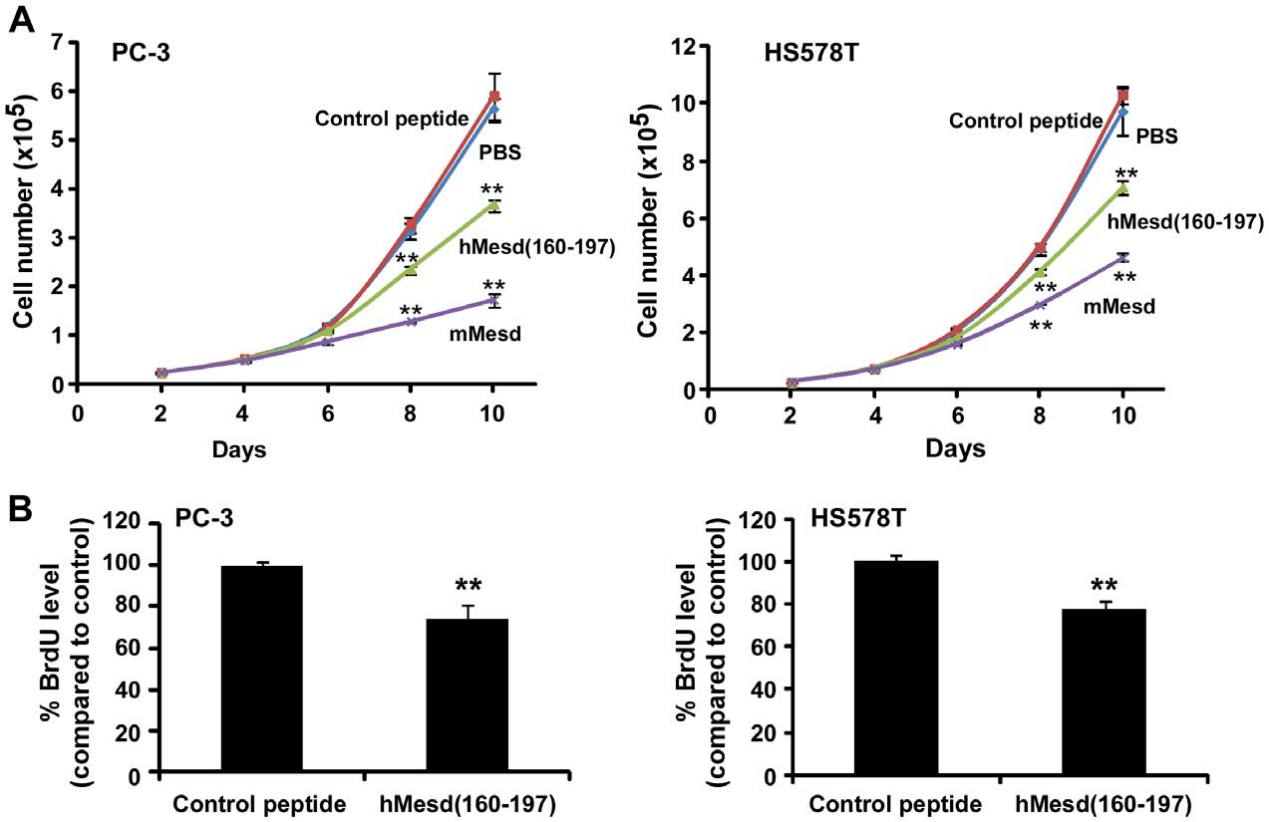
Mesd C-terminal region peptide inhibits prostate cancer PC-3 and breast cancer HS578T cell proliferation. (A) Cancer cells in 6-well plates were treated with mouse Mesd (mMesd, 2 µM), human Mesd peptide hMesd (160–197) (4 µM) or control peptide (4 µM) in RPMI-1640 medium containing 2% FBS for PC-3 cells or DMEM medium containing 2% FBS for HS578T for 10 days. The media were changed every other day, and cells were harvested and counted using the trypan blue exclusion assay. Values are averages of three determinations with the standard deviations indicated by error bars. ** *P*<0.01 compared to the cells treated with PBS or control peptide. (B) Cancer cells in T-25 flasks were treated with human Mesd peptide hMesd (160–197) (2 µM) or control peptide (2 µM) in the culture medium containing 2% FBS for 7 days. The media were changed every other day. The cells were then harvested and seeded into 96-well tissue culture plates at a density of 5000 cells/well with hMesd (160–197) (2 µM) or control peptide (2 µM) in the culture medium containing 10% FBS for 2 days. Cell proliferation was measured by BrdU proliferation ELISA. All the values are the average of six determinations with the s.d. indicated by error bars. **P<0.01 compared to cells treated with control peptide.

Having established that the Mesd C-terminal region peptide suppresses Wnt/β-catenin signaling in PC-3 and HS578T cells, we then examined the effect of the Mesd C-terminal region peptide on cancer cell proliferation. As seen in Figure 7, hMesd (160–197), but not the control peptide, mimicked Mesd protein and displayed inhibitory effect on PC-3 and HS578T cell proliferation in a time-dependent manner (Figure 7A). To confirm the inhibitory effect of the Mesd peptide on cancer cell proliferation, we performed BrdU incorporation assay after the cells were treated with peptides. It was found that the Mesd C-terminal region peptide decreased BrdU incorporation into PC-3 and HS578T cells (Figure 7B).

### Mesd protein and its C-terminal region peptide potentiate chemotherapy agent Adriamycin-induced cytotoxicity in PC-3 and HS578T cells

**Figure 8:**
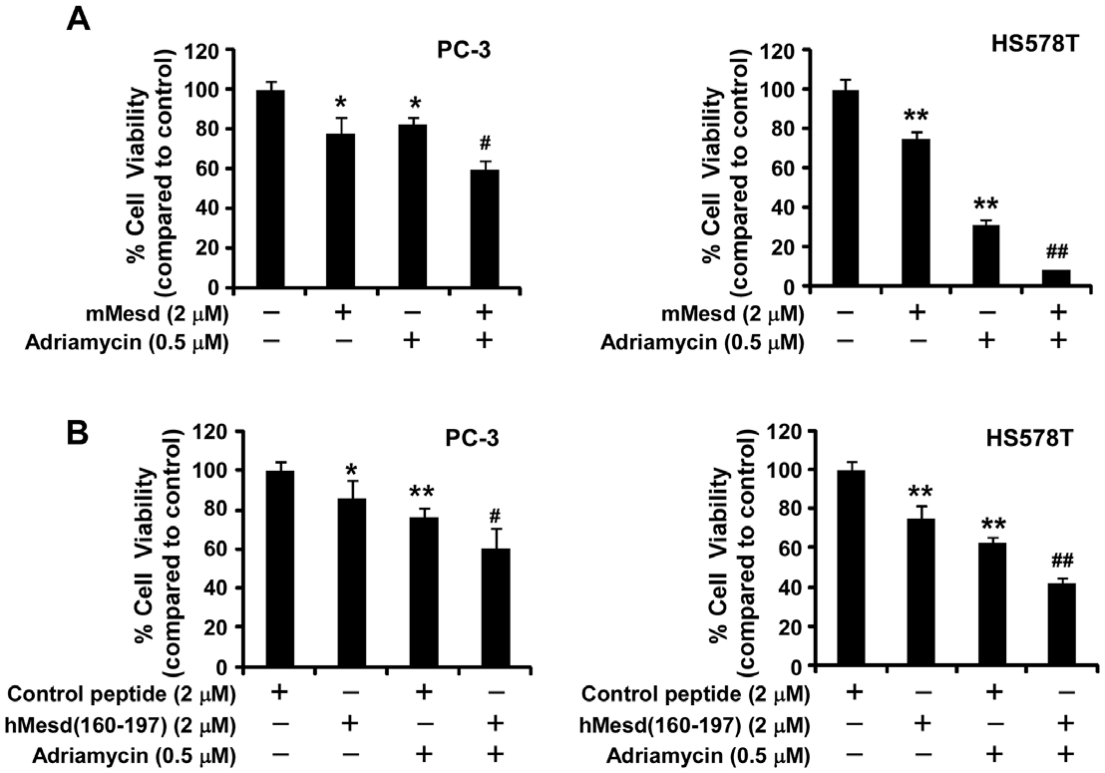
Mesd protein and its C-terminal region peptide potentiate chemotherapy agent Adriamycin-induced cytotoxicity in PC-3 and HS578T cells. (A) Cancer cells in T-25 flasks were treated with mouse Mesd (2 µM) in RPMI-1640 medium containing 2% FBS for PC-3 cells or DMEM medium containing 2% FBS for HS578T for 4 days. The media were changed every other day, and the cells were harvested and seeded into 96-well tissue culture plates at a density of 5000 cells/well with Mesd (2 µM) and/or Adriamycin (0.5 µM) in RPMI-1640 medium containing 10% FBS for PC-3 cells or DMEM medium containing 10% FBS for HS578T for 2 days. Cell viability was then measured by the Cell Titer Glo Assay system. (B) Cancer cells in T-25 flasks were treated with human Mesd peptide hMesd (160–197) (2 µM) or control peptide (2 µM) in the culture medium containing 2% FBS for 7 days. The cells were harvested and seeded into 96-well tissue culture plates at a density of 5000 cells/well with hMesd (160–197) (2 µM), control peptide (2 µM) and Adriamycin (0.5 µM) in the culture medium containing 10% FBS for 2 days. Cell viability was then measured by the Cell Titer Glo Assay system. All the values are the average of quadruple determinations with the s.d. indicated by error bars. *P<0.05, **P<0.01 compared to untreated cells or cells treated with control peptide. ^#^ *P*<0.05, ^##^ *P*<0.01 compared to cells treated with Mesd alone, hMesd (160–197) alone or Adriamycin alone.

Adriamycin is a common chemotherapy agent. We then tested whether Mesd protein and its C-terminal region peptide can increase chemotherapy agent Adriamycin-induced cytotoxicity in cancer cells. As seen in Figure 8, combination treatment caused more cytotoxicity in HS578T and PC-3 cells than individual agent treatment. For example, treatment of HS578T cells with Mesd protein (2 µM) alone and Adriamycin (0.5 µM) alone resulted in 25% and 69% inhibition of cell viability, respectively. However, when treated with Mesd protein plus Adriamycin, the cell viability of HS578T cells was reduced to 8% (Figure 8).

## Discussion

In our previous study, we found that the C-terminal region of Mesd (150–195), which is absent in sequences from invertebrates is necessary and sufficient for Mesd binding to mature LRP6 [12]. In the present study, we further demonstrated that Mesd C-terminal region peptides block Mesd binding to LRP5 at the cell surface. Importantly, we showed that there are two LRP5/6 binding sites within Mesd C-terminal region which contain several positively charged residues. By performing molecular dynamics simulations on Mesd C-terminal region peptides, we demonstrated that the two LRP5/6 binding sites within the Mesd C-terminal region peptide form a positive surface consisted of the solvent exposed sidechains of positively charged residues. Our findings are consistent with a previous study of the NMR structure of Mesd and the cell surface LRP6 binding of Mesd mutants, which showed that the positively charged residues within Mesd C-terminal region are important for Mesd binding to LRP6 at the cell surface [24].

Both LRP5 and LRP6 contain four β-propeller/epidermal growth factor (EGF) modules, named E1 to E4 from N- to C-terminal, and all the identified LRP5/6 extracellular ligands including Mesd bind to the β-propeller/EGF repeat modules [3], [37]–[41]. Recently, the crystal structures of LRP6 ectodomains E1–E2, E3–E4 and E1–E4 have been solved [42]–[44]. Chen et al. defined key elements of the interface between E3 and E4 which appear to be conserved for the interface between E1 and E2 and contributes to a compact platformlike architecture [43]. This LRP6 ectodomain architecture permits Mesd to engage E3 and E4 simultaneously in a pH-dependent manner [43]. There are about 19 vertebrate members of the Wnt family, and different Wnt proteins bind to distinct regions of LRP6. For example, Wnt 1, Wnt9a and Wnt10b bind exclusively within the LRP6 E1–E2 region *in vitro*, whereas Wnt3 and Wnt3a bind only to a fragment containing E3–E4 [39]–[41]. In our previous study, we reported that Mesd is a general Wnt inhibitor that blocks Wnt/β-catenin signaling induced not only by LRP6 E1–E2-binding Wnts but also by LRP6 E3–E4-binding Wnts [14]. In the present study, we further demonstrated that the Mesd C-terminal region peptide was able to block Wnt1, Wnt3 and Wnt10b-induced Wnt/β-catenin signaling in LRP5 or LRP6-expressing cells. Our results indicate that the Mesd C-terminal region peptide, like the full-length Mesd protein, is a general inhibitor of different Wnt proteins in Wnt/LRP signaling. In the present study, we also found that the Mesd C-terminal region peptide is less efficient against Wnt/LRP signaling and cancer cell proliferation than the full-length Mesd protein. This could be the lesser stability of the peptide. However, it has been found that Mesd protein and its peptide displayed a similar pharmacokinetics and bioavailability in mice [13].

Compelling evidence indicates that there is an abnormal up-regulation of the Wnt/β-catenin pathway in tumorigenesis of many types of cancer, and that dysregulation of Wnt/β-catenin signaling on the cell surface is associated with aberrant activation of this pathway in prostate and breast cancer [45], [46]. LRP6 expression is significantly up-regulated in prostate patients with metastatic disease compared to those without metastasis, and is associated with a significantly increased risk of recurrent disease [33]. Treatment of prostate cancer cells with Wnt3A CM or purified recombinant Wnt3A protein significantly enhanced cell growth and migration [31], [32], while treatment of prostate cancer cells with the LRP6 antagonist Dkk1 significantly inhibited cell growth and migration [32]. LRP6 is up-regulated in human triple negative breast cancer [13], [29], [30]. It was found that transcriptional knockdown of LRP6 in human triple negative breast cancer MDA-MB-231 cells significantly decreased Wnt/β-catenin signaling, cell proliferation, and tumor growth in vivo [13], and that blocking Wnt/β-catenin signaling by N-myc downstream regulated gene-1 (NDRG1), a tumor metastasis suppressor which interacts with LRP6 and represses Wnt/β-catenin signaling, led to drastic suppression of metastatic phenotypes of mammary tumor cells *in vitro* and *in vivo* [33]. Moreover, small molecule inhibitors targeting LRP6 were able to inhibit human breast and prostate cancer cell proliferation [47], [48], [49]. In our previous studies, we demonstrated that the full-length Mesd protein and the Mesd C-terminal region peptide suppressed MDA-MB-231 tumor growth [13], and that Mesd protein markedly inhibited Wnt/β-catenin signaling in prostate cancer PC-3 cells, and suppressed PC-3 cell proliferation in vitro and tumor growth in vivo [8], [14]. In the present study, we further demonstrated that the Mesd C-terminal region peptide, like Mesd protein, is able to suppress Wnt/β-catenin signaling in human prostate and breast cancer cells and inhibit cancer cell proliferation, although the full-length Mesd protein is more potent than its peptide. Moreover, we found that treatment of Mesd protein and its C-terminal region peptide significantly increased chemotherapy agent adriamycin-induced cytotoxicity in HS578T and PC-3 cells. Together, these results suggest that Wnt co-receptor LRP6 is a potential therapeutic target for cancer, and that Mesd protein and its peptide have therapeutic value in Wnt-dependent cancers.

## Supporting Information

Figure S1 Human Mesd C-terminal region peptide blocks Wnt/β-catenin signaling induced by LRP6, Wnt3A and Rspo1 in HEK293 cells. HEK293 cells in 24-well plates were transiently transfected with the LRP6 plasmid or the corresponding control vector, along with the Super8XTOPFlash luciferase construct and the β-galactosidase-expressing vector in each well. After 24 h incubation, cells were treated with Wnt3A CM (5%), Rspo1 (40 ng/ml), mouse Mesd protein (1 µM), human Mesd C-terminal region peptide hMesd(160–197) (8 µM) or control peptide (8 µM) at the indicated concentrations. The luciferase activity was then measured 24 h later with normalization to the activity of the β-galactosidase. Values are the average of triple determinations with the s.d. indicated by error bars. **P<0.01 compared to the control cells without Mesd and its peptide treatment.
(TIF)

Figure S2 Mesd blocks Wnt1- or Wnt10b-induced Wnt/β-catenin signaling in HEK293 cells. (A) HEK293 cells in 24-well plates were transiently transfected with the Wnt1 or Wnt10b plasmid along with the Super8XTOPFlash luciferase construct and β-galactosidase-expressing vector in each well. After 24 h incubation, cells were treated with mouse Mesd protein, human Mesd C-terminal region peptide hMesd (160–197) or control peptide at the indicated concentrations. The luciferase activity was then measured 24 h later with normalization to the activity of the β-galactosidase. Values are the average of triple determinations with the s.d. indicated by error bars. **P<0.01 compared to the control cells without Mesd or Mesd peptide treatment. (B) HEK293 cells in 6-well plates were transiently transfected with Wnt1 or Wnt10b plasmid or the corresponding control vector. After being incubated for 24 h, cells were treated with mouse Mesd, WTP or CP at the indicated concentrations for 24 h. The levels of cytosolic free β-catenin, and total cellular Wnt1, Wnt10b, β-catenin, LRP6, Axin2, cyclin D1 and phosphorylated LRP6 were then analyzed by Western blotting. Samples were also probed with the anti-actin antibody to verify equal loading.
(TIF)
