# Supplementary figures and images for: The C-Terminal Region Mesd Peptide Mimics Full-Length Mesd and Acts as an Inhibitor of Wnt/β-Catenin Signaling in Cancer Cells
Source: PLoS One. 2013 Feb 28;8(2):e58102. doi: 10.1371/journal.pone.0058102 (PMC3585277; doi:10.1371/journal.pone.0058102)

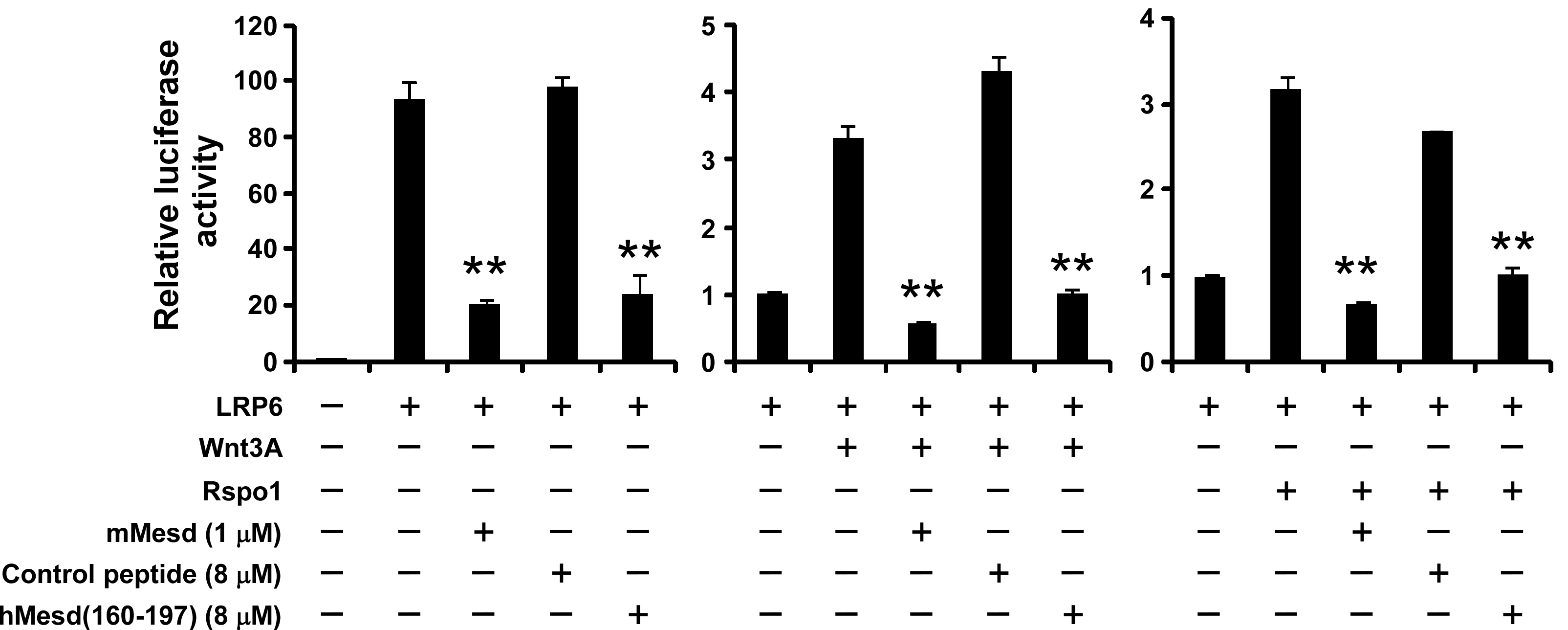

Supplement: Figure S1 — Human Mesd C-terminal region peptide blocks Wnt/β-catenin signaling induced by LRP6, Wnt3A and Rspo1 in HEK293 cells. HEK293 cells in 24-well plates were transiently transfected with the LRP6 plasmid or the corresponding control vector, along with the Super8XTOPFlash luciferase construct and the β-galactosidase-expressing vector in each well. After 24 h incubation, cells were treated with Wnt3A CM (5%), Rspo1 (40 ng/ml), mouse Mesd protein (1 µM), human Mesd C-terminal region peptide hMesd(160–197) (8 µM) or control peptide (8 µM) at the indicated concentrations. The luciferase activity was then measured 24 h later with normalization to the activity of the β-galactosidase. Values are the average of triple determinations with the s.d. indicated by error bars. **P<0.01 compared to the control cells without Mesd and its peptide treatment. (TIF) [file pone.0058102.s001.tif]

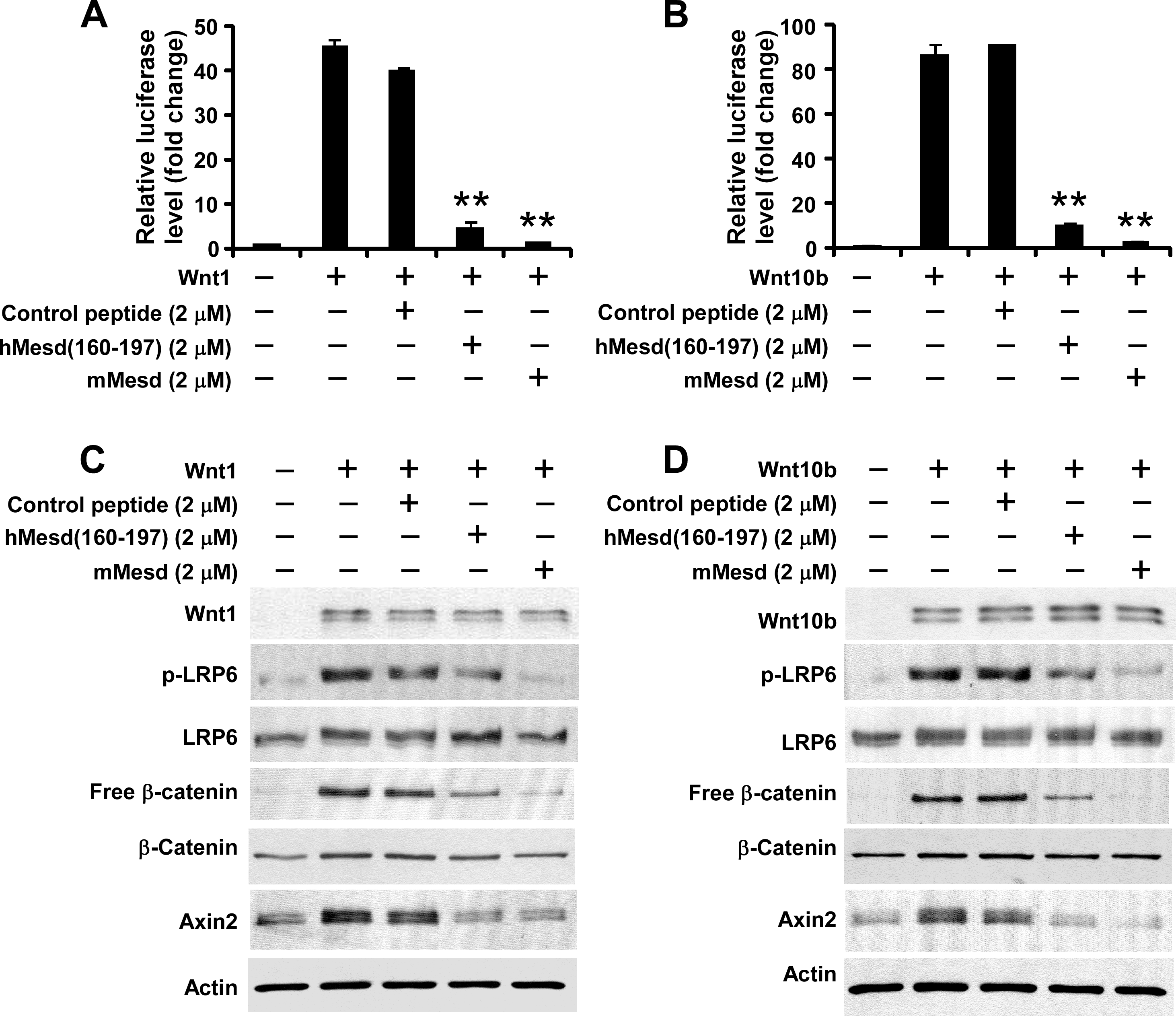

Supplement: Figure S2 — Mesd blocks Wnt1- or Wnt10b-induced Wnt/β-catenin signaling in HEK293 cells. (A) HEK293 cells in 24-well plates were transiently transfected with the Wnt1 or Wnt10b plasmid along with the Super8XTOPFlash luciferase construct and β-galactosidase-expressing vector in each well. After 24 h incubation, cells were treated with mouse Mesd protein, human Mesd C-terminal region peptide hMesd (160–197) or control peptide at the indicated concentrations. The luciferase activity was then measured 24 h later with normalization to the activity of the β-galactosidase. Values are the average of triple determinations with the s.d. indicated by error bars. **P<0.01 compared to the control cells without Mesd or Mesd peptide treatment. (B) HEK293 cells in 6-well plates were transiently transfected with Wnt1 or Wnt10b plasmid or the corresponding control vector. After being incubated for 24 h, cells were treated with mouse Mesd, WTP or CP at the indicated concentrations for 24 h. The levels of cytosolic free β-catenin, and total cellular Wnt1, Wnt10b, β-catenin, LRP6, Axin2, cyclin D1 and phosphorylated LRP6 were then analyzed by Western blotting. Samples were also probed with the anti-actin antibody to verify equal loading. (TIF) [file pone.0058102.s002.tif]
